# Trends in marriage and time spent single in sub-Saharan Africa: a comparative analysis of six population-based cohort studies and nine Demographic and Health Surveys

**DOI:** 10.1136/sti.2008.034249

**Published:** 2009-03-13

**Authors:** M Marston, E Slaymaker, I Cremin, S Floyd, N McGrath, I Kasamba, T Lutalo, M Nyirenda, A Ndyanabo, Z Mupambireyi, B Żaba

**Affiliations:** 1 London School of Hygiene and Tropical Medicine, London, UK; 2 Imperial College, London, UK; 3 Africa Centre for Health and Population Studies, University of KwaZulu-Natal, Durban, South Africa; 4 Medical Research Council/Uganda Virus Research Institute Uganda Research Unit on AIDS, Entebbe, Uganda; 5 Rakai Health Sciences Program, Kalisizo, Uganda; 6 Biomedical Research and Training Institute, Harare, Zimbabwe; 7 TAZAMA Project, National Institute for Medical Research, Mwanza, Tanzania

## Abstract

**Objectives:**

To describe trends in age at first sex (AFS), age at first marriage (AFM) and time spent single between events and to compare age-specific trends in marital status in six cohort studies.

**Methods:**

Cohort data from Uganda, Tanzania, South Africa, Zimbabwe and Malawi and Demographic and Health Survey (DHS) data from Uganda, Tanzania and Zimbabwe were analysed. Life table methods were used to calculate median AFS, AFM and time spent single. In each study, two surveys were chosen to compare marital status by age and identify changes over time.

**Results:**

Median AFM was much higher in South Africa than in the other sites. Between the other populations there were considerable differences in median AFS and AFM (AFS 17–19 years for men and 16–19 years for women, AFM 21–24 years and 18–19 years, respectively, for the 1970–9 birth cohort). In all surveys, men reported a longer time spent single than women (median 4–7 years for men and 0–2 years for women). Median years spent single for women has increased, apart from in Manicaland. For men in Rakai it has decreased slightly over time but increased in Kisesa and Masaka. The DHS data showed similar trends to those in the cohort data. The age-specific proportion of married individuals has changed little over time.

**Conclusions:**

Median AFS, AFM and time spent single vary considerably among these populations. These three measures are underlying determinants of sexual risk and HIV infection, and they may partially explain the variation in HIV prevalence levels between these populations.

Timing of sexual debut and subsequent marriage dynamics are considered important factors in the spread of HIV. The timing of marriage has been shown to be important as, at an individual level, early marriage for women has been associated with an increased risk of infection.^1^ Conversely, late marriage has been linked to the spread of HIV as an increase in the time between sexual debut and first marriage allows for the acquisition of more premarital sexual partners and thus a higher risk of acquiring HIV before first marriage,^2^ and a long time spent single and sexually active has been associated with a higher number of partners later in life.^3^

Patterns of formation and dissolution of marital unions also play an important role in the spread of HIV. Widowhood, divorce and separation have been associated with increased prevalence of HIV.^4^ ^5^ For example, in Manicaland, Zimbabwe, an exceptionally high prevalence of HIV was observed among widows and widowers (54% and 61%, respectively, in the 2001/3 survey).^6^ In the absence of discordancy, HIV is introduced to a marriage via extramarital partners.^7^ Furthermore, women in polygamous marriages have been shown to have a higher risk of acquiring HIV than those in monogamous marriages.^8^

Boerma *et al*^9^ found that the time spent sexually active and single was longer in Kisesa, Tanzania than in Manicaland, Zimbabwe, particularly for women. In both populations, men reported a longer time spent single than women. Subsequent analyses found that the average number of person-years spent sexually active before first marriage has not changed over time in Manicaland^10^ whereas, in Kisesa, an increase in time spent single has been observed,^3^ ^9^ although these and other behavioural differences were insufficient to explain the differences in the prevalence of HIV in these two settings.

This paper aims to examine trends in age at first sex (AFS), age at first marriage (AFM) and the length of time between first sex and first marriage in populations with generalised HIV epidemics. It also examines whether age-specific marital status distributions vary across these populations and over time.

## METHODS

Data from six population-based cohort studies which are part of the ALPHA network^11^ were analysed.^12^^–^17 In all cases the data are based on complete repeated censuses of the population. AFS and AFM were based on retrospectively reported ages. For respondents who participated in multiple surveys but gave inconsistent reports, corrections were made as reported in the site-specific papers in this supplement.^3^ ^10^ ^18^ ^19^ Time spent single and sexually active is defined as the time between first sex and first marriage and is referred to here as “time spent single”. For each cohort, two surveys were chosen as far apart as possible to compare marital status over time (table 1).

**Table 1:** Data overview for each cohort study

Study site (country)	Time period	Number reporting marital status(age 15–59 years)	Number (%) ever married(age 15–59 years)
*Men*			
Masaka (Uganda)	1997/8	4021	1947 (48.4)
	2006/7	5647	3189 (56.5)
Rakai (Uganda)	1999/00	1531	790 (51.6)
	2005/6	2057	1059 (51.5)
Kisesa (Tanzania)	1994/5	2648	1357 (51.3)
	2003/4	3590	2022 (56.3)
Karonga (Malawi)	2002/4	7293	4251 (58.3)
	2007/8	6994	2146 (61.9)
Manicaland (Zimbabwe)*	1998/00	2998	2363 (78.8)
	2003/5	3203	2450 (76.5)
Umkhanyakude (South Africa)†	2002	21584	4252 (19.7)
	2006	21023	3622 (17.2)
*Women*			
Masaka (Uganda)	1997/8	4267	3501 (82.1)
	2006/7	8580	7225 (84.2)
Rakai (Uganda)	1999/00	1706	1223 (71.7)
	2005/6	2858	2124 (74.3)
Kisesa (Tanzania)	1994/5	3042	2358 (77.5)
	2003/4	4539	3776 (83.2)
Karonga (Malawi)	2002/4	7853	6501 (82.8)
	2007/8	7704	3137 (81.8)
Manicaland (Zimbabwe)*	1998/00	3724	3250 (87.3)
	2003/5	4641	3970 (85.5)
Umkhanyakude (South Africa)†	2002	25164	6147 (24.4)
	2006	24115	5290 (21.9)

*Manicaland: 1998/2000 men aged 17–54 years, women aged 15–44 years, 2003/5 men and women aged 15–54 years.

†Umkhanyakude both periods: men aged 18–59 years, women aged 18–59 years.

Time periods indicate the times of the first and second surveys included in the analysis.

Demographic and Health Survey (DHS) data on AFS and AFM from Tanzania (1996, 1999, 2003, 2005), Uganda (1995, 2001) and Zimbabwe (1994, 1999, 2005)^20^ were also analysed and compared with cohort data from the same countries. Weights provided by the DHS were taken into account for point estimates and confidence intervals were calculated using bootstrapping, allowing for the strata and clusters in the survey design. Log rank tests were used to compare AFS, AFM and time spent single and sexually active for different birth cohorts.

In each study site, marriage is defined broadly to include religious, traditional and civil marriages. While some studies differentiated between married and non-marital cohabiting couples, we classified them as married for these analyses, with the exception of Umkhanyakude in KwaZulu Natal, South Africa, where non-marital cohabiting partnerships are not classed as married. For the purposes of this analysis, all other studies do not distinguish between non-marital cohabiting couples and married couples. A common classification of marital status was defined: never married, currently married and ex-married (the latter including divorced, widowed and separated individuals).

We use the term “monogamous marriage” to refer to marriage of two people rather than the absence of extramarital partners. Polygamous marriages were defined as marriages in which the man reported to have more than one spouse or the woman identified co-wives. It was possible to disaggregate polygamous from monogamous marriages for men and women in all sites with the exception of Manicaland (men only). In the Manicaland study, men are asked how many current spouses or regular partners they have. This broad definition was used to capture partnerships which are similar to marital unions but not formally defined as such. For the purposes of this analysis, polygamous and monogamous men in Manicaland are distinguished using this broader definition.

Life table methods were used to calculate median AFS, AFM and median time spent single for all DHS surveys and all cohort studies with the exception of Karonga (where these data were not available). This method uses retrospective reporting of the age at the event and age at time of survey to censor those who report never having sex or being unmarried.^21^ Results were stratified by 10-year birth cohort and analyses restricted to those born after 1950. Time spent single was not presented for the latest birth cohort (born 1980–9) as many participants reported never having had sex, biasing results towards those who have sex earlier who may have a different marital pattern to those with a later sexual debut. AFS and AFM are reported as age in years rather than as fractions of a year and, to allow for this, ages were distributed randomly within the year by generating a random number between 0 and 1 from a uniform distribution and adding it to the reported age. Integer values were used for median time spent single for most sites. Stata 10 (Statacorp, 2007) was used for all analyses.

## RESULTS

### Median age at first sex (AFS) and age at first marriage (AFM)

**Figure 1:**
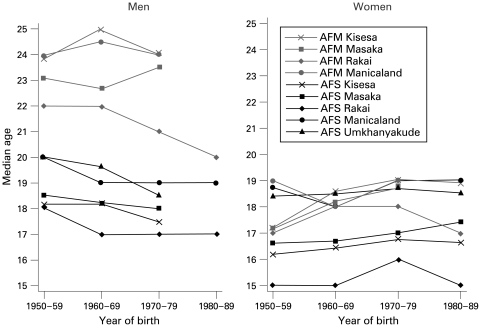
Median age at first sex (AFS) and age at first marriage (AFM) by sex, site and birth cohort. Note the median AFM for Umkhanyakude (not shown) for women was 35.1 years, 41.1 years and 38.5 years for birth cohorts 1950–9, 1960–9 and 1970–9, respectively.

Figure 1 shows the median AFS and AFM by birth cohort, sex and site. Median AFS was lower in the East African sites for both men and women. For men, median AFS has become lower in all sites when the earliest birth cohort (1950–9) is compared with the latest (1980–9). For women, median AFS in the East African sites lies between 15 and 17 years, much lower than in the Southern African sites where it lies between 18 and 19 years. AFS for women varied over time in different ways across the sites. In Manicaland there was an overall increase of more than 1 year between the 1960–9 cohort and the latest cohorts. In Masaka and Kisesa, median AFS appeared to have increased less, by around 6 months. In Umkhanyakude, which has the highest median AFS at around 18.5 years, there was almost no change over time.

In Umkhanyakude, median AFM was 44 years for men born in 1950–9 and could not be calculated for later male birth cohorts as less than 50% had married by the time of their last interview. For all other sites, the median AFM for men was below 25 years. AFM has remained at around 24 years for men in Manicaland. In Kisesa the median AFM increased and subsequently declined, the net effect being no overall change at around 24 years in the 1970–9 cohort. In Rakai, a considerable decline in median AFM has occurred from 22 years in the 1950–69 cohorts to 20 years for those in the 1980–9 birth cohort while, in Masaka, there is little evidence for change over time.

Median AFM for women varied between 17 and 19.5 years over time, apart from Umkhanyakude where median AFM was >35 years (data not shown). In Kisesa, AFM increased by almost 2 years between the earliest and latest birth cohorts, and there was a similar trend in Masaka where AFM increased by 1.5 years between the earliest and latest cohorts. Median AFM fluctuated in Rakai, increasing from 17 years for the 1950–9 birth cohort to 18 years for those born in 1960–79 and returning to 17 years for the 1980–9 birth cohort.

**Figure 2:**
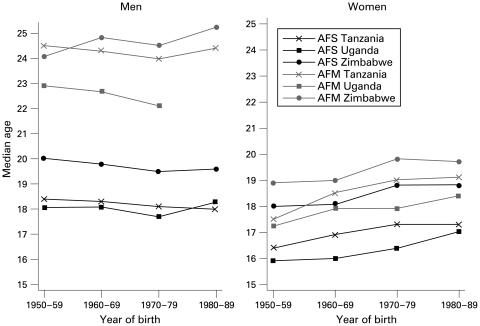
Median age at first sex (AFS) and age at first marriage (AFM) by sex, country and birth cohort using Demographic and Health Survey (DHS) data.

Nationally representative trends from DHS with respect to AFS correspond well with the trends observed in cohort studies from respective countries for men and women (fig 2). Marriage levels and trends were also similar when comparing the sites with the corresponding national DHS. However, for men in Masaka there was an overall increase in AFM whereas the Uganda DHS showed a decrease between the 1950–9 and 1970–9 birth cohorts from 22.9 years (95% CI 22.3 to 23.5) to 22.1 years (95% CI 21.7 to 22.4) (p<0.001). Also, women in Manicaland had a slightly younger median AFM compared with the Zimbabwe DHS.

### Time spent single and sexually active (time between first sex and first marriage)

**Figure 3:**
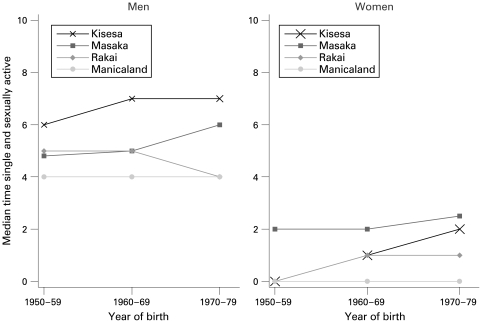
Median time (in years) spent single and sexually active by sex, site and 10-year birth cohort.

For men, the net effect of changes in AFS and AFM on the median time spent single was an increase of around 1 year for men in Kisesa and Masaka (fig 3). In Rakai, time spent single decreased by 1 year between the earliest and latest cohorts, while in Manicaland there was no change in time spent single between the birth cohorts considered here. For the 1970–9 birth cohort, the median time spent single ranged from 4 years in Manicaland and Rakai to 7 years in Kisesa.

For women, across all the study sites the median time spent single was much shorter than for men, and in Manicaland it was zero for all birth cohorts as 70% of women reported the same AFM and AFS.^10^ There was an overall increase in the median time spent single among women in the three East African sites. In Kisesa and Rakai this increase was around 1 year, while in Masaka it was only about 6 months. For the 1970–9 birth cohort, the median time spent single ranged from 0 years in Manicaland to 2.5 years in Masaka.

**Figure 4:**
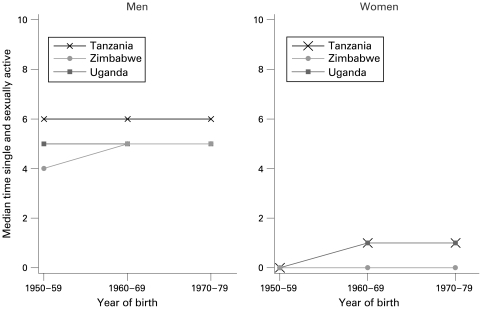
Median years spent single and sexually active by sex, country and 10-year birth cohort using Demographic and Health Survey (DHS) data.

For women in Rakai and Manicaland, the median number of years spent single was the same as those obtained from the DHS for Uganda and Zimbabwe, respectively (fig 4). The Tanzanian and Ugandan DHS indicated a shorter median time spent single than that observed for the cohort studies in Kisesa and Masaka. The median number of years that women in the 1970–9 cohort spent single (2 years) was 1 year longer in Kisesa than the DHS reported for Tanzania (1 year (95% CI 0.84 to 1.17)) and 2.5 times longer (2.5 years) in Masaka than reported in the Uganda DHS (1 year (95% CI 0.69 to 1.33)). Many more women in these DHS reported AFS to be the same as AFM than in the cohort surveys.

### Trends in marital status

#### Age-specific trends

**Figure 5:**
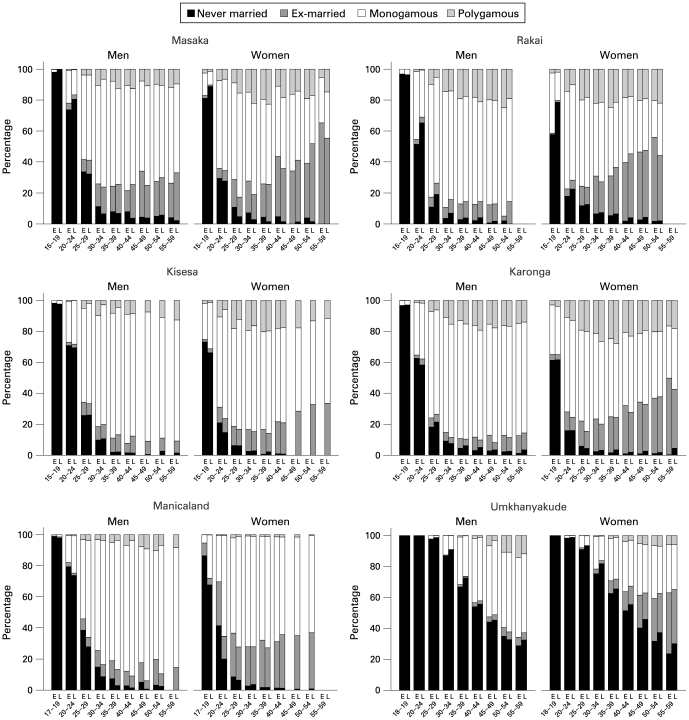
Marital status by sex and earlier (E) and later (L) time period. Note that the youngest age group is different in Umkhanyakude and Manicaland. In Manicaland, for men polygamy uses a broader definition which includes regular partners.

Figure 5 shows marital status for the two selected surveys by age group, sex and site. As expected, the proportion of married subjects increased with age, as did the proportion of ex-married individuals. One of the most striking differences across the sites is the low proportion of married subjects in Umkhanyakude compared with the other sites, with over 50% of men and women never married in the 40–44-year-old age group compared with <4% of women and 5% of men in the other sites. Excluding Umkhanyakude, the percentage of women never married in the latest period ranged from 15% (Kisesa) to 28% (Masaka) for those aged 20–24 years and from 3% (Kisesa and Masaka) to 7% (Rakai) for those aged 30–34 years. For men the range was from 62% (Karonga) to 80% (Masaka) for those aged 20–24 years and from 7% (Karonga and Masaka) to 11% (Kisesa) for those aged 30–34 years.

The proportion of people ex-married (widowed, divorced or separated) varied across the sites. Excluding Umkhanyakude, the percentage of ex-married subjects in the 40–44 year age groups ranged from 5% (Karonga) to 22% (Masaka) for men and from 20% (Kisesa) to 41% (Rakai) for women. The proportion of ex-married people was consistently much higher for women than for men.

For approximately 1% of married individuals in Umkhanyakude, it was not possible to classify their marriage as polygamous or monogamous. For women in the later period in Masaka, less than 1% of the population could not be classified into monogamous or polygamous groups; for the earlier period it was less than 2%, except for the 40–44 year age group where 4% could not be classified. For men, a higher proportion of the population was known to be married but not classified as monogamous or polygamous, at around 4% for both time periods in those aged >30 years and up to 6% for younger age groups. For all other sites, less than 1% of married individuals could not be classified as monogamous or polygamous. Those known to be married but not defined as monogamous or polygamous were classified as monogamous for this analysis.

Polygamous marriages are most common in Karonga and Rakai. For women, the proportion in polygamous marriages described an inverted U-shape with an increase with age followed by a decrease at around 30–39 years of age as the number of those widowed, divorced or separated increased. This pattern was particularly apparent in Karonga and Masaka. For men, the proportion in polygamous marriages tended to increase steadily with age. In Karonga, 13% of women aged 30–39 years were in polygamous marriages, which was 45% of all married women in this age group.

The proportion of men in monogamous marriages remained at around 80% for Kisesa after the age of 29 years compared with approximately 70%, 65%, 60% and <50% in Karonga, Rakai, Masaka and Umkhanyakude, respectively. For women the levels of monogamy were lower with the highest proportion of monogamously married women in Manicaland at around 70% for those aged 25–39 years, decreasing to around 60% for women aged 40–54 years. In Umkhanyakude the proportion of individuals in monogamous marriages was very low (approximately 30% in those aged 35–59 years). A similar pattern was seen in Karonga, Rakai and Masaka, with around 40% of all women in monogamous marriages after the age of 35 years.

#### Trends over time

The proportion of people never married has increased over time in Umkhanyakude and, to a lesser extent, in Rakai and Karonga. In Kisesa, with the longest time between the comparison surveys (9 years), a decrease in polygamous marriage for men and women was observed, especially at younger ages, indicating that fewer people are now entering polygamous marriages. Among those aged 30–34 years in 1994/5, 23% of married women reported being in polygamous marriages but, by 2004/5, this had fallen to 15%. A slight decrease in polygamy was also observed in Rakai. In Karonga and Masaka the opposite occurred for both men and women, with a small increase in polygamy between 2002/4 and 2007/8 in Karonga and over the 9-year period from 1997/8 to 2006/7 in Masaka. In Umkhanyakude and Manicaland, where polygamy is less common, small decreases were observed.

The proportion of people ex-married (widowed, divorced or separated) varied slightly over time. In Karonga, Manicaland and Umkhanyakude a slight decrease for men over 30 was observed, although this could be due to the decrease in those getting married, as mentioned above. In Kisesa there was an increase in ex-married men. Among women in Kisesa there was a slight decrease for those aged over 30 years, but in the other sites there did not seem to be any large changes or trends by age over time. Overall, the proportion of people married in each age group seemed to have changed little between the two surveys compared for each site.

## DISCUSSION

Similar to previous findings,^9^ there were large differences in AFS, AFM and time spent single and sexually active between countries. Reported AFS and AFM were lower in the East African settings than in the Southern African settings. In Umkhanyakude a very different pattern was observed, with lower marriage rates and later marriage than in all the other sites. Common to all sites was an older AFM for men than for women and a longer time spent single. The cohort results were broadly similar to those found in the DHS, echoing the findings comparing AFS measured in cohort studies and in the DHS in Uganda.^22^ The strongest differences were among women in Masaka and in the 1970–9 cohort in Kisesa where there was a longer median time spent single owing to a slightly lower median AFS than in the Tanzania DHS, and in Masaka a slightly higher median AFM compared with the Uganda DHS. These differences may be explained by the different demographic structure of the sites, since the DHS is designed to be nationally representative.

In all study sites apart from Umkhanyakude, cohabiting is indistinguishable from marriage in terms of local perceptions and this is reflected in data gathering, so that date of first marriage and first cohabitation are treated as though they were synonymous. In Umkhanyakude a distinction is made between marriage and cohabitation; however, data on age at first cohabitation with partner are not available so the two statuses cannot be grouped together and treated in the same way for survival analysis calculation of AFM or cohabitation. However, we have solid evidence that cohabitation and marriage occur later in Umkhanyakude than in the other sites, based on current status data. Grouping together married women and those who are cohabiting with their sexual partners in Umkhanyakude, the percentage of all women who were married or cohabiting at the age of 25–29 years was only 14% in 2006^23^ compared with 72–85% in the other study sites at their later time reference points, and 47% at the age of 45–49 years compared with 53–71% in the other sites. Although we can be certain that AFM and/or cohabitation is much higher in Umkhanyakude, the difference between age at marriage in Umkhanyakude and age at marriage and/or cohabitation in the other sites is somewhat higher than would be seen were it possible to compare like with like, because of the exclusion of the relatively small group of women in Umkhanyakude for whom cohabitation is a prelude to marriage.

**Take-home messages**

- There is considerable variation in age at first sex (AFS), age at first marriage (AFM) and time spent single and sexually active in Eastern and Southern Africa.
- Time spent single and sexually active is longer for men than women; sex differences in median time range from 2 to 5 years.
- Estimates of AFS and AFM based on repeated measures in longitudinal studies are similar to corresponding estimates from national Demographic and Health Surveys.

It is important to look at the median time spent single in conjunction with median AFS and AFM as this measure can conceal underlying changes in AFS and AFM. Overall, there has been an increase in the time spent sexually active before marriage, which may increase the potential for accumulating a higher number of premarital sexual partners^3^ ^24^ and has been associated with more frequent extramarital sex later in life.^3^ ^24^ ^25^

AFS and AFM are subject to reporting biases. Recent studies have found evidence for inconsistent reporting of AFS and AFM in Masaka, Umkhanyakude, Manicaland^18^ and in Kisesa.^3^ However, these studies also found that these inconsistencies had little effect on the overall trends in AFS and AFM if simple corrections were made. As discussed elsewhere, combining data from repeated cross sections in a cohort study should be less prone to biases than in the DHS.^21^ ^22^ Both the DHS and the cohort studies show similar results, which gives us confidence that these results are reliable.

AFM may be difficult for an individual to define because in many African societies marriage is a process rather than a discrete event,^26^ ^27^ and consequently individuals may find it hard to report their exact age at the time they were married. Some may take the AFM to be the start of sexual relations while others may take it to be further along the marriage process, and this difference in perspective may have cultural roots. In most studies the definition of marriage included both formal and informal marriages, but in Umkhanyakude only formal (ie, traditional and civil marriages) were included. In Umkhanyakude a decline in marriage among the Zulu population has been observed for decades.^28^ ^29^ Some of the reasons suggested for the substantial declines in marriage include the effect of apartheid era policies such as labour migration and family separation,^30^ as well as specific aspects of Zulu marriage and childbearing traditions.^31^

With the exception of Umkhanyakude where cohabiting partners were not included, the proportion of people married after 25 years of age was very high and has not changed much over time. There was a much higher proportion of widowed, divorced and separated women at all ages compared with men, with the difference increasing with age. This is caused in part by earlier widowhood among women (because of higher male mortality and older age of husbands), as well as a higher likelihood of remarriage among men. In Zambia, 85% of previously married men remarried compared with 52% of women.^32^ Also, it may be because husbands tend to die before their wives as they are usually older. It is surprising not to see an overall increase in the proportion of ex-married individuals due to an increase in widowhood from HIV, and an increase in divorce due to reduced tolerance of extramarital sexual activity. This could be because the time interval for these analyses for many of the sites is not long enough to capture this effect or it may be hidden by remarriage. Polygamous unions are still common, especially in East Africa.

Overall levels of HIV prevalence were highest in Umkhanyakude (27% for resident women and 14% for men in the 2003/4 period)^33^ and Manicaland (21% for women and 17% for men in 2003/5)^34^ and lowest in Kisesa (11% for women and 9% for men in 2003/4)^35^ and Masaka (8% overall in 2004/5).^36^ This order of levels of prevalence has been the same for at least the last 10 years. Time spent single and sexually active was longest in Umkhanyakude for men and women, followed by Kisesa and Masaka, and shortest in Manicaland. If the length of time spent single and sexually active is an important contributor to driving and sustaining the epidemic, then we might expect those places where the median time spent single and sexually active is longest to have the highest HIV prevalence. The observed differences in HIV prevalence between Umkhanyakude, Kisesa and Masaka are compatible with this, but the results from Manicaland are not. This is because AFS, AFM and time spent single are *underlying* rather than proximal determinants of HIV transmission in the conceptual framework for understanding the determinants of HIV dynamics.^37^ Proximal determinants—including number and type of partnerships, frequency of partnership change and behaviour within partnerships (such as condom use)—can modify the effects of the underlying determinants which can be thought of as “enabling circumstances” rather than direct risks. Better understanding of the associations between AFS, AFM and time spent single and HIV risk will require analyses of behaviour data that include information on proximal determinants.

In summary, these data illustrate differences in AFS and AFM among countries in East and Southern Africa. Apart from Umkhanyakude where both men and women spend a long time single and sexually active, most women get married soon after becoming sexually active while men usually spend several years single so, for men in particular, this could increase their exposure to HIV and other sexually transmitted infections.
